# Primary cutaneous amyloidosis associated with autoimmune hepatitis-primary biliary cirrhosis overlap syndrome and Sjögren syndrome: A case report

**DOI:** 10.1097/MD.0000000000010004

**Published:** 2018-02-23

**Authors:** Xin Yan, Jinglan Jin

**Affiliations:** Department of Hepatology, The First Hospital of Jilin University, Changchun, Jilin, China.

**Keywords:** amyloidosis, immunity, overlap syndrome, Sjögren syndrome

## Abstract

**Rationale:**

Primary cutaneous amyloidosis (PCA) is a localized skin disorder characterized by the abnormal deposition of amyloid in the extracellular matrix of the dermis. The association between PCA and other diseases, although rare, has been documented for various autoimmune diseases. PCA associated with autoimmune hepatitis-primary biliary cirrhosis (AIH-PBC) overlap syndrome and Sjögren syndrome (SS) has not been previously reported in the literature.

**Patient concerns:**

A 50-year-old woman presented with progressive abnormal liver enzyme levels and was referred to our department.

**Diagnoses:**

Due to the patient's symptoms, laboratory test results, radiographic findings, and pathologic results, she was diagnosed with PCA associated with AIH-PBC overlap syndrome and SS.

**Interventions:**

She was subsequently treated with a combination of ursodeoxycholic acid (UDCA), prednisone, and azathioprine.

**Outcomes:**

While this treatment can achieve therapeutic success, it cannot prevent complications from cirrhosis. This patient remains alive but experienced an emergent gastrointestinal hemorrhage.

**Lessons:**

While we acknowledge that this is a single case, these findings extend our knowledge of immunological diseases associated with PCA and suggest a common, immune-mediated pathogenic pathway between PCA, AIH-PBC overlap syndrome, and SS. After 12 years of follow up, clinical manifestations have developed, and these autoimmune diseases have progressed. The combination of UDCA, prednisone, and azathioprine can achieve therapeutic success but cannot prevent disease progression. Routine follow up for this patient is necessary to document disease progression.

## 1 Introduction

Primary cutaneous amyloidosis (PCA) is a localized skin disorder characterized by the abnormal deposition of amyloid in the extracellular matrix of the dermis.^[1]^ Autoimmune hepatitis-primary biliary cirrhosis (AIH-PBC) overlap syndrome refers to the coexistence of these conditions and is defined as a distinct autoimmune liver disease.^[2]^ Sjögren syndrome (SS) is a multisystem autoimmune disease characterized by the hypofunction of salivary and lacrimal glands, as well as systemic multiorgan manifestations like skin, lung, kidney, etc.^[3]^ The occurrence of PCA with other diseases is rare, but it has been documented in combination with various autoimmune diseases, including primary biliary cirrhosis,^[4]^ systemic sclerosis,^[5]^ scleroderma.^[6]^ This article describes a case of PCA associated with AIH-PBC overlap syndrome and SS that has not been previously published in the literature. Herein, a common immune-mediated pathogenic pathway is hypothesized and a therapeutic regimen is provided for reference.

## 2 Case report

A 50-year-old woman was followed up in our hospital since 2005 because of abnormal liver enzyme levels.

In spring 2005, the patient's γ-glutamyltransferase (γ-GT) levels were 177 U/L following a cesarean section in the department of gynecology and obstetrics of our hospital. However, she received no treatment at that time.

**Figure 1:**
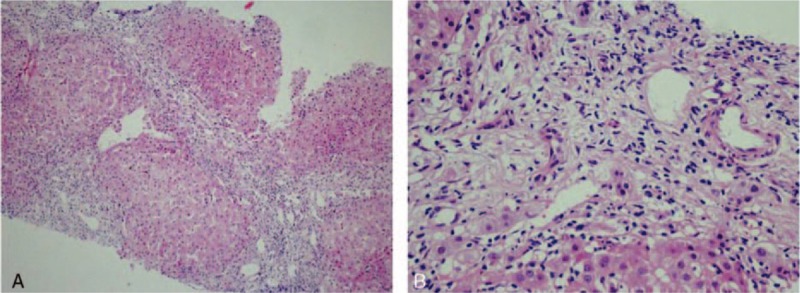
A and B: Liver biopsy results show severe interface hepatitis and nonsuppurative destructive cholangitis.

In 2008, she had elevated levels of γ-GT (241 U/L) and alkaline phosphatase (ALP, 133 U/L) without jaundice, pruritic papules, or other symptoms. She was referred to the hepatology service of the same hospital during that period. She reported no family history of hepatic disease, no fever, no current medications, and no consumption of alcohol, blood transfusions, or contact with individuals with hepatitis. No positive signs were observed on physical examination. A laboratory examination excluded infection with hepatitis A, B, or C. An abdominal ultrasound showed the thickness of the spleen to be 41 mm. Determination of the presence of serum antinuclear antibodies (ANA) was positive (1:1000) by immunofluorescence; there was no indication of the presence of the M2 fraction of antimitochondrial antibody (AMA-M2, 9 RU/mL) by enzyme-linked immunosorbent assay (ELISA). The patient was subjected to a percutaneous liver biopsy, which showed nonsuppurative destructive cholangitis with severe interface hepatitis (Fig. 1A and B). The patient was diagnosed with AIH-PBC overlap syndrome but refused to take prednisolone because of its potential adverse effects, such as moon face and weight gain. The patient was treated instead with ursodeoxycholic acid (UDCA, 750 mg/d) with improvement of symptoms and cholestatic enzyme levels.

In 2012, the patient came to our hospital and presented with dry eyes, dry mouth, and pruritus. ANA levels were also positive (1:3200) by immunofluorescence and AMA-M2 levels were elevated (159 RU/mL) by ELISA. Liver function tests revealed that γ-GT (496 U/L) and ALP (292 U/L) levels were significantly increased. The patient was treated with a combination of UDCA (750 mg/d) and intravenous hydroprednisone (30 mg/d) for 10 days; methylprednisolone tablets (6 mg/d) were administered orally for 3 months.

**Figure 2:**
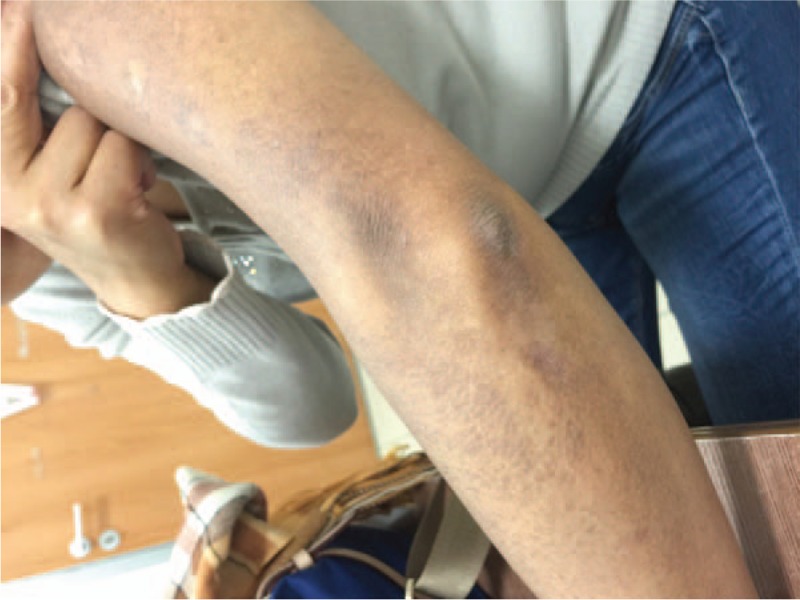
Pruritic papules with brownish pigmentation of the limbs.

**Figure 3:**
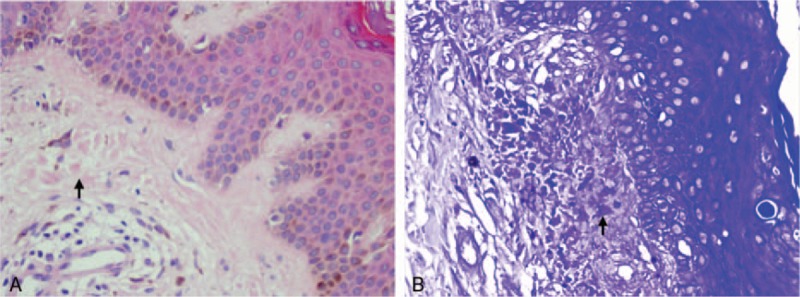
Skin biopsy results revealed an amyloid deposit after staining with hematoxylin and eosin (A) and crystal violet (B).

**Figure 4:**
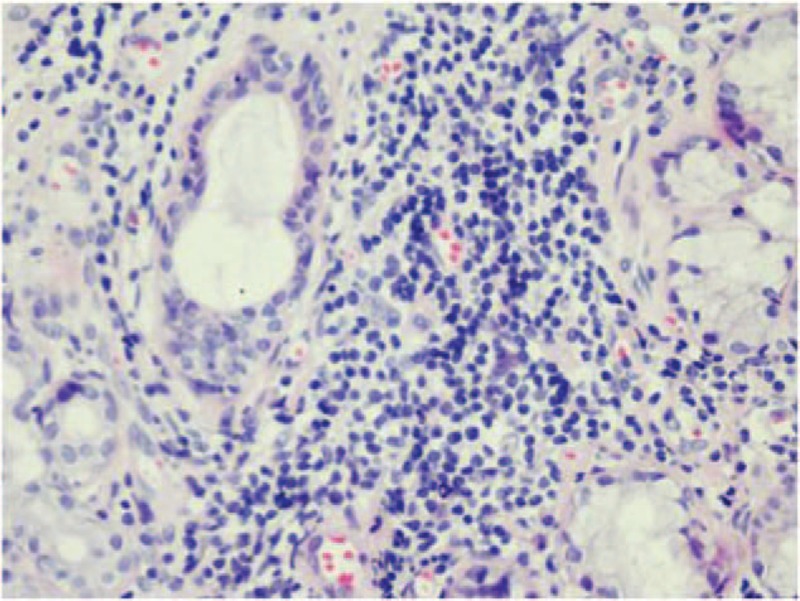
The histology of the labial gland shows focal lymphocytic sialadenitis, with a focus score ≥1 focus/4 mm^2^.

In 2014, the patient came to our hospital because of pruritic papules with brownish pigmentation of the trunk and limbs (Fig. 2). The patient also reported abdominal distension, edema of the lower limbs, and dry eyes and mouth during the past month. A physical examination showed jaundice and a painless liver with a thick edge 14 cm from the right costal margin. Liver function tests revealed γ-GT levels of 459 U/L and ALP levels of 340 U/L. ANA levels (1:3200) were the same as in 2012, but AMA-M2 levels were again elevated (200 RU/mL) by ELISA. A CT scan of the liver showed evidence of cirrhosis, a thick spleen (62 mm), and portal hypertension with collateral circulation. A skin biopsy revealed an amyloid deposit after staining with hematoxylin and eosin (H&E), and crystal violet (Fig. 3A and B). Histology of the labial gland showed focal lymphocytic sialadenitis, with a focus score ≥1 focus/4 mm^2^ (Fig. 4), according to the current European–American consensus criteria for the classification of SS. The patient was diagnosed with AIH-PBC overlap syndrome combined with cutaneous amyloidosis and SS. We advised the patient to undergo liver transplantation, but she refused. The patient was treated with prednisolone acetate (20 mg/d), azathioprine (50 mg/d), and UDCA (750 mg/d).

**Figure 5:**
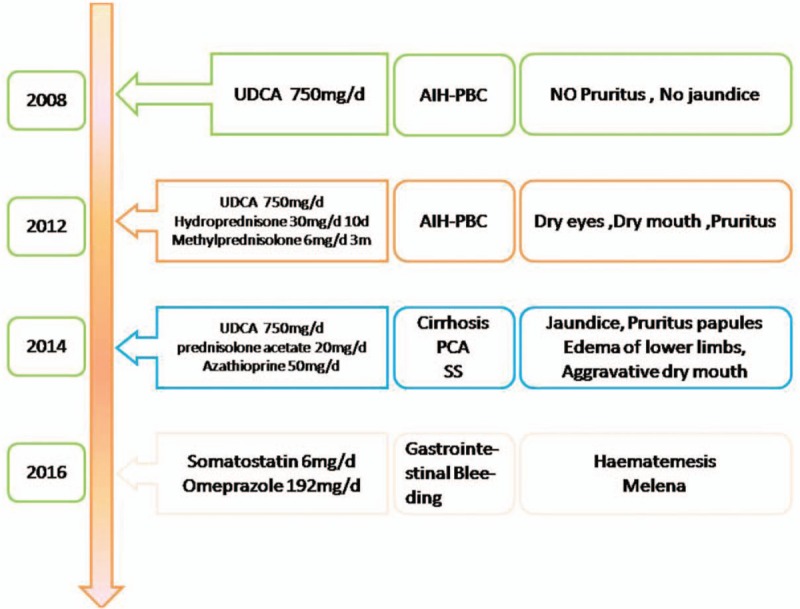
Timeline of interventions, diagnoses, and symptoms.

**Figure 6:**
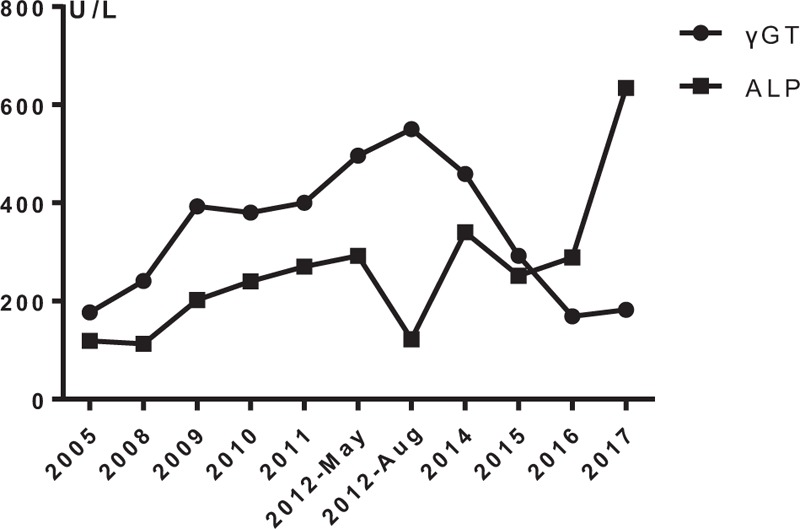
Evolution of γ-glutamyltransferase (γ-GT, normal 5–54 U/L) and alkaline phosphatase (ALP) (normal 11–112 U/L) levels from 2005 to 2017.

In 2016, with the progression of cirrhosis, the patient came to our hospital after experiencing hematemesis and melena for 3 days, but she refused to have esophageal variceal ligation; as a result, the patient was treated with somatostatin and omeprazole with improvement of both symptoms and cholestatic enzyme levels. The patient has been closely followed since that time. Figure 5 shows a timeline, detailing the patient's diagnoses, interventions, symptoms, and outcomes. Figure 6 shows the evolution of γ-GT and ALP levels over time. This case report was approved by the ethics committee of The First Hospital of Jilin University, Changchun, China, and written informed consent was obtained.

## 3 Discussion

PCA is a relatively rare condition characterized by amyloid deposition exclusively in the dermis without involving the internal organs. It can be divided into 3 general categories: lichen, macular, and nodular forms.^[7]^ The histopathology of cutaneous amyloidosis shows eosinophilic hyaline material in the papillary dermis by H&E staining, which can be confirmed by Congo red staining.^[8]^

In hepatology, overlapping features between AIH and PBC, primary sclerosing cholangitis, or indeterminate cholestasis, so-called overlap syndromes, usually progress into cirrhosis and/or liver failure without adequate treatment.^[9]^ AIH-PBC overlap syndrome has been reported at frequencies ranging from approximately 10% to fewer than 2% of patients with AIH or PBC alone.^[10]^ The Paris criteria, which are commonly used to define the presence of PBC with features of AIH, are endorsed by the European Association for the Study of the Liver (EASL).^[11]^ The symptoms of patients with overlap syndrome are often nonspecific and include fatigue, malaise, nausea anorexia, and abdominal pain.^[12]^

SS is a chronic inflammatory autoimmune disease with an obscure origin; it attacks the lacrimal and salivary glands specifically. Sicca symptoms are the hallmarks of this disease, which may also present with various organ manifestations.^[13]^ A high occurrence in women, B cell hyperactivity expressed as hypergammaglobulinemia, and the presence of several serum autoantibodies, as well as activation of type I interferon pathways, are disease characteristics also shared by other systemic autoimmune disorders, implying a common underlying pathogenic mechanism.^[14]^

The pathogenesis of PCA is still unknown; however, PCA could be concomitant with various immune diseases, implying a common underlying immune-mediated mechanism.^[15]^ Associations described so far include primary biliary cirrhosis, systemic sclerosis, scleroderma, Kimura disease, and SS.^[4–6,16,17]^ A pathogenic relationship between PCA and keratinocyte apoptosis has been hypothesized.^[18]^ Kamada et al^[19]^ reported that bilirubin caused damage to cultured keratinocytes, which may lead to cutaneous amyloidosis. Damage of bile duct epithelial cells is thought to be involved in the pathogeneses of cholestatic disease.^[20]^

Perhaps some common structures found in both keratinocytes and bile duct epithelial cells are targeted by autoantibodies. In addition, some authors have surmised that amyloidosis and SS have a common pathogenic pathway, such that the permeability of plasma cells in certain tissues (for instance, the skin and salivary glands) can generate light-chain immunoglobulins that cause amyloid deposits.^[21]^ Interestingly, AIH-PBC overlap syndrome is related to changes in plasma cells and immunoglobulin production.^[22]^ It is possible that PCA, AIH-PBC overlap syndrome, and SS share common immunological characteristics. Further investigations of the pathogeneses of PCA, AIH-PBC overlap syndrome, and SS would be worthwhile.

Controlling pruritus is important for the management of PCA. The treatments for PCA include retinoids, corticosteroids, cepharanthin, amitriptyline, colchicine, tacrolimus, cyclophosphamide, cyclosporine, vitamin D3 analogs, dimethyl sulfoxide, capsaicin, menthol, surgical modalities, hydrocolloid dressings, phototherapy, and lasers.^[23]^ We currently lack randomized, controlled data that could inform the optimal treatment of patients with AIH-PBC overlap syndrome,^[24]^ which presently involves a combination of anticholestatic (UDCA) and immunosuppressive therapies (corticosteroids and/or azathioprine).^[12]^ Liver transplantation is a life-saving treatment for patients with end-stage disease.^[25]^

## 4 Conclusions

Although PCA is benign,^[26]^ the symptoms, liver function index, biochemical response rate, and histologic changes observed in our patient after treatment with a combination of UDCA, prednisone, and azathioprin were all improved. As this patient has entered the decompensation period of liver cirrhosis, her prognosis is poor.

## References

[1] Cai D Li Y Zhou C Comparative proteomics analysis of primary cutaneous amyloidosis. Exp Ther Med 2017;14:3004–12. 28912854 10.3892/etm.2017.4852 PMC5585729

[2] Yokokawa J Saito H Kanno Y Overlap of primary biliary cirrhosis and autoimmune hepatitis: characteristics, therapy, and long term outcomes. J Gastroenterol Hepatol 2010;25:376–82. 19817953 10.1111/j.1440-1746.2009.06018.x

[3] Shiboski CH Shiboski SC Seror R 2016 American College of Rheumatology/European League against rheumatism classification criteria for primary Sjögren's syndrome. Arthritis Rheumatol 2017;69:35–45. 27785888 10.1002/art.39859 PMC5650478

[4] Kikuchi N Sakai E Nishibu A Primary localized cutaneous amyloidosis in patients with scleroderma. Acta Derm Venereol 2010;90:326–7. 20526567 10.2340/00015555-0855

[5] Tafarel JR Lemos LB Oliveira PM Cutaneous amyloidosis associated with primary biliary cirrhosis. Eur J Gastroenterol Hepatol 2007;19:603–5. 17556910 10.1097/MEG.0b013e32811ec024

[6] Ogiyama Y Hayashi Y Kou C Cutaneous amyloidosis in patients with progressive systemic sclerosis. Cutis 1996;57:28–32. 8620682

[7] Borowicz J Gillespie M Miller R Cutaneous amyloidosis. Skinmed 2011;9:96–100. quiz 101. 21548513

[8] Mehrotra K Dewan R Kumar JV Primary cutaneous amyloidosis: a clinical, histopathological and immunofluorescence study. J Clin Diagn Res 2017;11:WC01–5. 10.7860/JCDR/2017/24273.10334 PMC5620892 28969251

[9] Beuers U Rust C Overlap syndromes. Semin Liver Dis 2005;25:311–20. 16143946 10.1055/s-2005-916322

[10] Bonder A Retana A Winston DM Prevalence of primary biliary cirrhosis-autoimmune hepatitis overlap syndrome. Clin Gastroenterol Hepatol 2011;9:609–12. 21440668 10.1016/j.cgh.2011.03.019

[11] European Association for the Study of the Liver. Electronic Address E E E, European Association for the Study of The L. EASL Clinical Practice Guidelines: the diagnosis and management of patients with primary biliary cholangitis. J Hepatol 2017;67:145–72. 28427765 10.1016/j.jhep.2017.03.022

[12] Boberg KM Chapman RW Hirschfield GM Overlap syndromes: the International Autoimmune Hepatitis Group (IAIHG) position statement on a controversial issue. J Hepatol 2011;54:374–85. 21067838 10.1016/j.jhep.2010.09.002

[13] Stefanski AL Tomiak C Pleyer U The diagnosis and treatment of Sjogren's Syndrome. Dtsch Arztebl Int 2017;114:354–61. 28610655 10.3238/arztebl.2017.0354 PMC5471601

[14] Mavragani CP Mechanisms and new strategies for primary Sjogren's Syndrome. Annu Rev Med 2017;68:331–43. 28099084 10.1146/annurev-med-043015-123313

[15] Dahdah MJ Kurban M Kibbi AG Primary localized cutaneous amyloidosis: a sign of immune dysregulation? Int J Dermatol 2009;48:419–21. 19335432 10.1111/j.1365-4632.2009.03799.x

[16] Mazori DR Femia AN Primary cutaneous nodular amyloidosis in association with Sjogren's syndrome. Joint Bone Spine 2016;83:350. 26494586 10.1016/j.jbspin.2015.03.015

[17] Danno K Horio T Miyachi Y Coexistence of Kimura's disease and lichen amyloidosus in three patients. Arch Dermatol 1982;118:976–80. 7149753

[18] Ono K Fujimoto E Fujimoto N In vitro amyloidogenic peptides of galectin-7: possible mechanism of amyloidogenesis of primary localized cutaneous amyloidosis. J Biol Chem 2014;289:29195–207. 25172508 10.1074/jbc.M114.592998 PMC4200272

[19] Kamada N Yoneyama K Togawa Y Toxic epidermal necrolysis with severe hyperbilirubinemia: complete re-epithelialization after bilirubin reduction therapies. J Dermatol 2010;37:534–6. 20536667 10.1111/j.1346-8138.2009.00770.x

[20] Carey EJ Ali AH Lindor KD Primary biliary cirrhosis. Lancet 2015;386:1565–75. 26364546 10.1016/S0140-6736(15)00154-3

[21] Konishi A Fukuoka M Nishimura Y Primary localized cutaneous amyloidosis with unusual clinical features in a patient with Sjogren's syndrome. J Dermatol 2007;34:394–6. 17535407 10.1111/j.1346-8138.2007.00296.x

[22] Gonzalez-Moreno EI Camara-Lemarroy CR Borjas-Almaguer DO Cutaneous amyloidosis associated with autoimmune hepatitis-primary biliary cirrhosis overlap syndrome. Ann Hepatol 2015;14:416–9. 25864224

[23] Weidner T Illing T Elsner P Primary localized cutaneous amyloidosis: a systematic treatment review. Am J Clin Dermatol 2017;18:629–42. 28342017 10.1007/s40257-017-0278-9

[24] Lindor KD Gershwin ME Poupon R Primary biliary cirrhosis. Hepatology 2009;50:291–308. 19554543 10.1002/hep.22906

[25] Rust C Beuers U Overlap syndromes among autoimmune liver diseases. World J Gastroenterol 2008;14:3368–73. 18528934 10.3748/wjg.14.3368 PMC2716591

[26] Kaltoft B Schmidt G Lauritzen AF Primary localised cutaneous amyloidosis–a systematic review. Dan Med J 2013;60:A4727. 24192243

